# A population-based validation study of the 8th edition UICC/AJCC TNM staging system for cutaneous melanoma

**DOI:** 10.1186/s12885-022-09781-0

**Published:** 2022-07-01

**Authors:** Matthew C. Hynes, Paul Nguyen, Patti A. Groome, Yuka Asai, Meaghan E. Mavor, Tara D. Baetz, Timothy P. Hanna

**Affiliations:** 1 School of Medicine, Queen’s University, Kingston, ON Canada; 2 Nuffield Department of Population Health, University of Oxford, Oxford, UK; 3 ICES Queen’s, Queen’s University, Kingston, ON Canada; 4 Division of Cancer Care and Epidemiology, Department of Public Health Sciences, Queen’s Cancer Research Institute, Queen’s University, 10 Stuart Street, 2nd Level, Kingston, ON K7L 3N6 Canada; 5 Division of Dermatology, Department of Medicine, School of Medicine, Faculty of Health Sciences, Queen’s University, Kingston, ON Canada; 6 Department of Oncology, Queen’s University, Kingston, ON Canada

**Keywords:** Melanoma, Skin neoplasms, Neoplasm staging, Ontario, Survival analysis, Prognosis, Cohort studies

## Abstract

**Background:**

The 8^th^ edition UICC/AJCC TNM8 (Tumour, Nodes, Metastasis) melanoma staging system introduced several modifications from the 7^th^ edition (TNM7), resulting in changes in survival and subgroup composition. We set out to address the limited validation of TNM8 (stages I-IV) in large population-based datasets.

**Methods:**

This retrospective cohort-study included 6,414 patients from the population-based Ontario Cancer Registry diagnosed with cutaneous melanoma between January 1, 2007 and December 31, 2012. Kaplan–Meier curves estimated the melanoma-specific survival (MSS) and overall survival (OS). Cox proportional hazard models were used to estimate adjusted hazard ratios for MSS and OS across stage groups. The Schemper-Henderson measure was used to assess the variance explained in the Cox regression.

**Results:**

In our sample, 21.3% of patients were reclassified with TNM8 from TNM7; reclassifications in stage II were uncommon, and 44.1% of patients in stage III were reclassified to a higher subgroup. Minimal changes in MSS curves were observed between editions, but the stage IIB curve decreased and the stage IIIC curve increased. For TNM8, Stage I (*n* = 4,556), II (*n* = 1,206), III (*n* = 598), and IV (*n* = 54) had an estimated 5-year MSS of 98.4%, 82.5%, 66.4%, and 14.4%, respectively. Within stage III, IIIA 5-year MSS was 91.7% while stage IIID was 23.5%. HRs indicated that TNM8 more evenly separates subgroups once adjusted for patient- and disease-characteristics. The variance in MSS explained by TNM7 and TNM8 is 18.9% and 19.7%, respectively.

**Conclusion:**

TNM8 performed well in our sample, with more even separation of stage subgroups and a modest improvement in predictive ability compared to TNM7.

**Supplementary Information:**

The online version contains supplementary material available at 10.1186/s12885-022-09781-0.

## Introduction

Melanoma is the eighth most common cancer among Canadians with an estimated 7,800 new cases in 2019 [1]. Melanoma is the most fatal form of skin cancer, and the incidence among Canadians has risen over several decades. In recent years, the incidence of melanoma has begun to stabilize in countries including Canada, Australia, the U.S., and New Zealand [1–3].

The American Joint Committee on Cancer and the Union for International Cancer Control both released the 8^th^edition (AJCC8 and UICC8) of the Melanoma Staging Manual in 2017 [4, 5]. The updated classification and staging guidelines clarified clinical terminology and introduced several changes to the TNM (Tumour, Node, Metastasis) system. Within T categories, Breslow thickness measurements are recorded to one decimal place, T1a and T1b are redefined, and mitotic rate is no longer a staging criterion. Within N categories, there is further stratification by the number of tumor-involved lymph nodes. The eighth edition also introduced a fourth stage III subgrouping (IIID) and restructured the T and N combinations of each respective stage III subgroup [4]. TNM8 refers to the 8^th^ edition staging criteria for melanoma shared by AJCC8 and UICC8.

Changes to the AJCC/UICC staging system were made following the creation and analysis of the International Melanoma Database and Discovery Platform (IMDDP). The IMDDP is an institutional series including > 46,000 patients with stage I-III cutaneous melanoma from five countries (Australia, Greece, Italy, Spain, U.S.A.). The resulting TNM8 changes demonstrated superior separation of stage subgroup Kaplan–Meier curves and a 15–29 percentile point increase in survival for stages IIIA/B/C [4].

The majority of previous TNM8 validation studies are institutional series [6–13]. Large population-based studies evaluating the performance of the entire TNM8 (stages I-IV) are lacking. These are important to represent the full spectrum of patients with melanoma. For example, age distribution or the presence of important pathologic prognostic factors such as ulceration may not be adequately represented in institutional populations. Moreover, treatments outside of highly-specialized tertiary care centres may vary, affecting melanoma-specific survival [6, 7, 12].

In this paper, we assess the 8^th^ edition UICC/AJCC (TNM8) staging system for cutaneous melanoma in a Canadian population-based sample using the Ontario Cancer Registry. We detail the extent of subgroup reclassification, changes to stage-specific MSS and OS, adjusted hazard ratios across stage groups, and the amount of survival variance explained by TNM8.

## Materials & methods

### Data sources and linkage

Data utilized in this study were obtained from administrative datasets at ICES. ICES, formerly known as the Institute for Clinical Evaluative Sciences, is an independent, non-profit research institute funded by an annual grant from the Ontario Ministry of Health and the Ministry of Long-Term Care. As a prescribed entity under Ontario’s privacy legislation, ICES is authorized to collect and use health care data for the purposes of health system analysis, evaluation, and decision support. Secure access to these data is governed by policies and procedures that are approved by the Information and Privacy Commissioner of Ontario. All personal identifying information is removed at ICES, and an anonymous unique identifier, the ICES Key Number (IKN), is generated for each patient.

The Ontario Cancer Registry (OCR) is a population-based tumor registry administered by Cancer Care Ontario (CCO). The OCR captures > 95% of newly diagnosed cutaneous melanoma in Ontario, Canada [14, 15]. The OCR passively collects cancer data on the > 14 million residents of Ontario through pathology reporting, hospital records, CCO treatment centres, and death records. The OCR records diagnostic information required for staging. Demographic information, including date of birth, sex, and address, was obtained from the Registered Persons Database (RPDB), a repository for all Ontario residents who are eligible for Ontario Health Insurance Plan (OHIP). All information on all deaths, including the cause of death, registered in Ontario is provided by the Office of the Registrar General-Deaths (ORGD) data. Records of acute hospital inpatient and day surgery admissions and discharges were collected from the Canadian Institute for Health Information Discharge Abstract Database and Same Day Surgery (CIHI DAD and SDS) data. Records of radiation and systemic therapy were collected within Activity Level Reporting (ALR) data. Records of intravenous drugs approved for delivery in Ontario were documented in the New Drug Funding Program (NDFP) and drug benefits for all adults aged 65 + and those receiving social assistance were collected from the Ontario Drug Benefit (ODB) program. These datasets were linked using unique encoded identifiers and analyzed at ICES.

### Patient selection

**Fig. 1:**
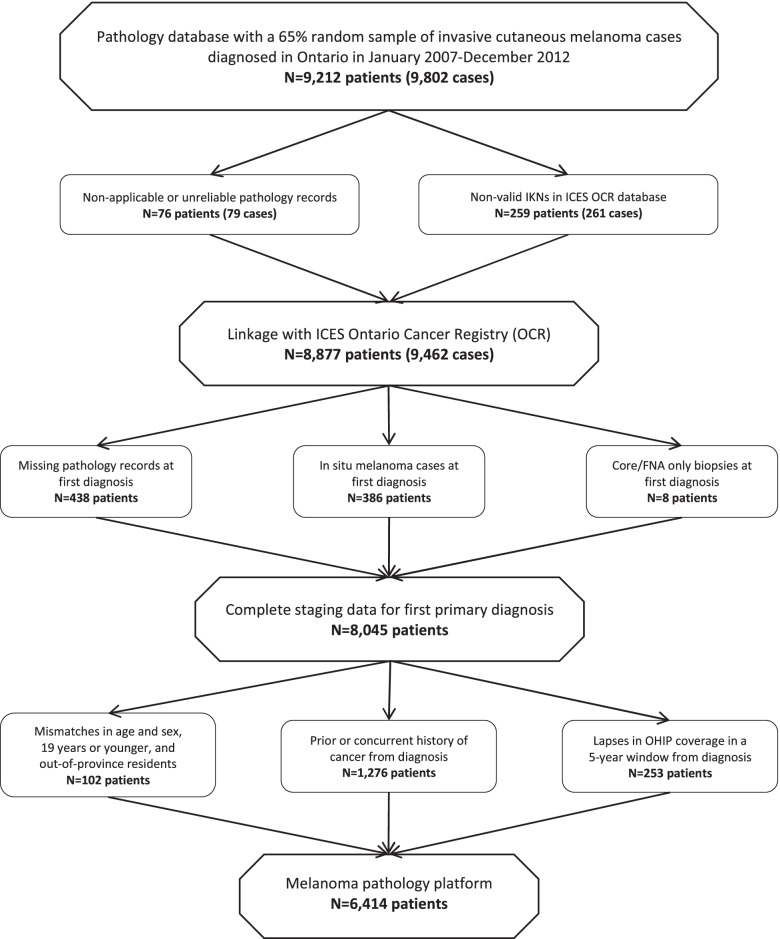
Identification of invasive cutaneous melanoma patients and their pathology records in Ontario from January 1, 2007 to December 31, 2012

A retrospective population-based cohort was derived from a 65% random sample of cutaneous melanoma recorded in the OCR and diagnosed between January 1, 2007 and December 31, 2012. The 65% random sample was a convenience sample, with the sample size calculated for the power requirements of the parent study investigating melanoma treatment outcomes according to stage [16–18]. Eligible participants were residents of Ontario, > 19 years in age, diagnosed with invasive cutaneous melanoma, and had no previous history or concurrent cancer (Fig. 1). Patients were followed up to 5 years from diagnosis, or until death, whichever occurred first.

Regarding disease characteristics, patients were excluded for prior or concurrent cancer diagnosis, in situ melanoma at first diagnosis, and core/FNA biopsies only, due to concerns of confounding disease, incomplete OCR reporting, and inaccurate staging, respectively. To preserve data accuracy, patients were excluded for missing pathology records, unreliable pathology records, lapses in OHIP coverage within 5-years of diagnosis, non-valid IKN in the OCR, and demographic incompatibility. This study was approved by the Queen’s University Health Sciences and Affiliated Teaching Hospitals Research Ethics Board (EPID-425–13). This study followed the Strengthening the Reporting of Observational Studies in Epidemiology reporting guideline for cohort studies.

### Classification of independent variables

Data on the following disease characteristics were collected: histological subtype, Breslow thickness, mitotic rate, ulceration, location, and other attributes required for TNM staging (Table 1). Patients were staged according to the TNM 7^th^ edition (TNM7) and TNM8 criteria and combinations. For the T-category, “missing” ulceration results were treated as “absent” ulceration to provide the minimal staging information.

**Table 1:** Patient and disease characteristics

**Eligible participants**	6,414	**Stage at Diagnosis**	
**Age**		**TNM7**	
Median (IQR)	61 (49–73)	IA	2,634 (41.1%)
**Sex**		IB	1,914 (29.8%)
Female	3,097 (48.3%)	Stage I total	4,548
Male	3,317 (51.7%)	IIA	529 (8.3%)
**Breslow Thickness (mm)**		IIB	405 (6.3%)
Median (IQR)	0.8 (0.5–2.0)	IIC	280 (4.4%)
0 to < 0.8	2,887 (45.0%)	Stage II total	1,214
0.8 to 1	803 (12.5%)	IIIA	172 (2.7%)
> 1 to 2	1,153 (18.0%)	IIIB	219 (3.4%)
> 2 to 4	786 (12.3%)	IIIC	207 (3.2%)
> 4	732 (11.4%)	IIID	-
Missing	53 (0.8%)	Stage III total	598
**Location**		IV	54 (0.8%)
Arm or shoulder	1,611 (25.1%)	**TNM8**	
Head and neck	1,168 (18.2%)	IA	3,692 (57.6%)
Leg or hip	1,470 (22.9%)	IB	864 (13.5%)
Trunk	2,128 (33.2%)	Stage I total	4,556
Other, or missing	37 (0.6%)	IIA	524 (8.2%)
**Ulceration**		IIB	402 (6.3%)
Present	1,080 (16.8%)	IIC	280 (4.4%)
Absent	4,899 (76.4%)	Stage II total	1,206
Missing	435 (6.8%)	IIIA	112 (1.8%)
**Histological Subtype**		IIIB	122 (1.9%)
Melanoma, NOS	2,041 (31.8%)	IIIC	325 (5.1%)
Nodular melanoma	781 (12.2%)	IIID	39 (0.6%)
Lentigo maligna melanoma	441 (6.9%)	Stage III total	598
Superficial spreading melanoma	2,684 (41.9%)	IV	54 (0.8%)
Acral lentiginous melanoma	98 (1.5%)	**T category**	
Other	369 (5.8%)	**TNM7**	
**Mitotic rate (per mm**^**2**^**)**		T1a	2,653 (41.4%)
< 1	1,785 (27.8%)	T1b	1,053 (16.4%)
≥ 1	3,150 (49.1%)	T2a	992 (15.5%)
Missing	1,479 (23.1%)	T2b	186 (2.9%)
**Residential Area**		T3a	459 (7.2%)
Urban (RIO < 10)	3,862 (60.2%)	T3b	338 (5.3%)
Suburban (10 ≤ RIO < 40)	1,809 (28.2%)	T4a	242 (3.8%)
Rural (RIO ≥ 40)	677 (10.6%)	T4b	491 (7.7%)
Missing	66 (1.0%)	**TNM8**	
**Neighbourhood income quintile**		T1a	2,910 (45.4%)
1 (Lowest)	879 (13.7%)	T1b	833 (13.0%)
2	1,079 (16.8%)	T2a	967 (15.1%)
3	1,248 (19.5%)	T2b	186 (2.9%)
4	1,442 (22.5%)	T3a	452 (7.1%)
5 (Highest)	1,749 (27.3%)	T3b	334 (5.2%)
Missing	17 (0.3%)	T4a	241 (3.8%)
**Elixhauser comorbidity index**		T4b	491 (7.7%)
Mean ± SD	0.3 ± 0.9	**N category**	
0	5,347 (83.4%)	**TNM7**	
1	558 (8.7%)	Nx	5,351 (83.4%)
2–3	362 (5.6%)	N0	441 (6.9%)
4 +	147 (2.3%)	N1a	224 (3.5%)
**Adjuvant/palliative systemic therapy**		N1b	37 (0.6%)
Any systemic therapy	413 (6.4%)	N2a	105 (1.6%)
**Adjuvant systemic therapy**		N2b	22 (0.3%)
Interferon	372 (5.8%)	N2c	119 (1.9%)
**Palliative systemic therapy**		N3	115 (1.8%)
Any systemic therapy	51 (0.8%)	**TNM8**	
Dacarbazine	26 (0.4%)	Nx	5,351 (83.4%)
Carbo-Taxol	8 (0.1%)	N0	441 (6.9%)
Temozolomide	≤ 5 (≤ 0.1%)	N1a	224 (3.5%)
BRAF and/or MEK	12 (0.2%)	N1b	37 (0.6%)
Immunotherapy	≤ 5 (≤ 0.1%)	N1c	119 (1.9%)
**Adjuvant/palliative radiotherapy**		N2a	105 (1.6%)
Any body site	196 (3.1%)	N2b	22 (0.3%)
Brain-directed treatment	37 (0.6%)	N2c	30 (0.5%)
Non-brain-directed treatment	181 (2.8%)	N3a	16 (0.3%)
		N3b	26 (0.4%)
		N3c	43 (0.7%)

Age and sex were determined from the RPDB. Area-level income quintiles were determined based on quintile rankings of neighbourhood average income within each census metropolitan area or census agglomeration. Rurality was measured with the 2008 Rurality Index for Ontario (RIO) scale, ranging from 0 to 100, and categorised as urban (RIO < 10), suburban (10 ≤ RIO < 40), and rural (RIO ≥ 40). Comorbidity was based on the Elixhauser comorbidity index using CIHI DAD and SDS data with a 5-year lookback from diagnosis. Adjuvant and palliative-intent systemic therapy was measured using ALR, NDFP, and ODB data from 1-year following diagnosis. Radiation treatment was also based on ALR data within 1-year following diagnosis.

### Classification of dependant variables

Cause-of-death information was available up to December 31, 2017. Therefore, complete follow-up to death or five years was available for all included patients. Melanoma-specific survival (MSS) and overall survival (OS) were measured up to 5 years from the date of diagnosis, or until death, if the event had occurred earlier. A five-year cut-off was chosen to avoid bias associated with incomplete follow-up to 10 years for most of the cohort. MSS captured underlying deaths due to melanoma defined with ICD-10 diagnosis code ‘C43’ in the ORGD data while OS captured all causes of death.

### Statistical analysis

Kaplan–Meier methods were first used to generate the MSS and OS estimates and pointwise 95% confidence limits by the stage groups. Log-rank tests were used to evaluate the differences of MSS and OS amongst the stage groups. Then, Cox proportional hazard regression analyses were performed with adjustments of the stage group hazard ratios for patient and disease factors. The Schemper-Henderson measure was used to calculate the percent of the variance explained by stage in the Cox regression, which allowed data from censored participants to be used in the assessment of cure prediction [19–21]. For our analyses, two-sided statistical significance was determined using a *p*-value < 0.05.

Statistical analyses were conducted using the SAS software version 9.4 (SAS Institute Inc., Cary, NC).

## Results

### Baseline characteristics

Of the 9,212 patients initially selected, 2,798 were excluded; patients were most often excluded for prior or concurrent cancer diagnosis (1,276), missing pathology records (438), and in situ melanoma at first diagnosis (386) (Fig. 1). In total, 6,414 patients met the inclusion criteria for analysis. The median age at diagnosis was 61 years (interquartile range [IQR] 49–73) and just over half of the patients were male (51.7%). The most common subtypes of melanoma were superficial spreading (41.9%) and unspecified (31.8%). Melanoma was most often located on the trunk (33.2%) with a median Breslow thickness of 0.8 mm (IQR 0.5–2.0) and an ulceration rate of 16.8% (Table 1).

### Stage grouping and reclassification

Of the 6,414 melanomas in our study, 1,365 (21.3%) received a different stage subgroup upon reclassification from TNM7 to TNM8. Within stages I and II, all movement was to lower stage subgroups: 1,056 from IB to IA, and 11 from IIA/B to a lower stage or subgroup. The remaining reclassifications occurred within the stage III subgroupings. Of the 598 stage III melanomas in TNM7, 298 (49.8%) were reclassified in TNM8, 264 (44.1%) of which moved to a higher subgroup. Stage IIIB was the most common subgroup in TNM7 (219 cases; 36.6%) whereas stage IIIC was the most common subgroup in TNM8 (325 cases; 54.3%). Stage IIIC had the highest conservation between TNM7 and TNM8 with 75.8% of cases remaining IIIC. Stage IIIB had the lowest conservation, with only 24.7% of cases remaining unchanged. All 39 TNM8 stage IIID cases were originally TNM7 stage IIIC (see Additional file 1).

### Melanoma-specific survival

**Fig. 2:**
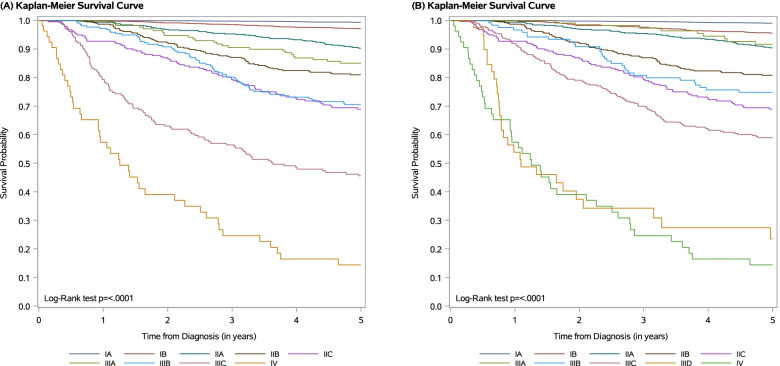
Melanoma-specific survival (MSS) for the TNM (A) 7^th^ edition and (B) 8^th^ edition

The Kaplan–Meier MSS curves according to the TNM 7^th^ and 8^th^ edition can be found in Figs. 2A and B, respectively. Patients were followed for an average of 4.60 years, with 5,916 patients censored at 5 years for the MSS analysis. Notable changes in MSS between TNM7 and TNM8 were present for stage IB and stages IIIA/B/C disease. Compared to TNM7, TNM8 stage IB experienced a modest decrease in 2-year and 5-year MSS, while TNM8 stages IIIA/C experienced a 3–16 percentile point increase in 2-year and 5-year MSS.

Several of the TNM8 Kaplan–Meier curves (Fig. 2B) cross. 2-year and 5-year MSS for stage IIIA was greater than stage IIA, IIB, and IIC. TNM8 stage IIIB also had a 2-year and 5-year MSS greater than stage IIC. MSS decreased for each stage from IA-IIC and from IIIA-IIID (Table 2). Among stage III, there was wide separation of subgroups. Stage IIIA had the best survival followed by IIIB, IIIC, and IIID. The confidence limits for stage IIID and stage IV overlap at the 2-year and 5-year point estimates.

**Table 2:** 2-year and 5-year melanoma-specific survival and 95% confidence interval (CI) for each stage of the 7^th^ and 8^th^ edition TNM melanoma staging system

	Melanoma-specific Survival							
	TNM7				TNM8			
Stage	2-year	95% CI	5-year	95% CI	2-year	95% CI	5-year	95% CI
IA	99.9%	99.6–100.0%	99.4%	99.0–99.6%	99.8%	99.6–99.9%	99.1%	98.7–99.3%
IB	99.2%	98.6–99.5%	97.1%	96.2–97.8%	98.5%	97.4–99.1%	95.6%	94.0–96.8%
IIA	96.9%	95.0–98.1%	90.3%	87.4–92.6%	97.1%	95.2–98.2%	90.4%	87.5–92.7%
IIB	92.2%	89.1–94.5%	81.0%	76.5–84.6%	92.2%	89.0–94.5%	80.8%	76.4–84.5%
IIC	86.6%	81.8–90.1%	69.0%	62.7–74.4%	86.6%	81.8–90.1%	69.0%	62.7–74.4%
IIIA	94.7%	90.1–97.2%	85.0%	78.6–89.6%	98.2%	93.1–99.6%	91.7%	84.6–95.6%
IIIB	90.8%	86.0–93.9%	70.5%	63.8–76.3%	90.9%	84.1–94.8%	74.8%	66.0–81.7%
IIIC	63.0%	55.9–69.3%	45.6%	38.3–52.5%	79.1%	74.2–83.2%	59.0%	53.1–64.3%
IIID	-	-	-	-	37.4%	22.3–52.4%	23.5%	10.8–39.0%
IV	39.0%	25.7–52.1%	14.4%	6.3–25.6%	39.0%	25.7–52.1%	14.4%	6.3–25.6%

For the OS analysis, the Kaplan–Meier curves followed similar trends, with 5,398 patients censored at 5 years (see Additional file 2). There was an improved 2-year and 5-year OS for TNM8 stage IIIA/B/C compared to TNM7 stage IIIA/B/C. Similarly, the 2-year and 5-year OS for TNM8 stage IIIA was greater than stage IIA, IIB, and IIC. The 2-year and 5-year OS for stage IIID was again overlapping with stage IV (see Additional file 3).

### Cox regression model & hazard ratios

The Cox regression model for the TNM8 staging system demonstrated that hazard ratios (HRs) for stages IA-IIB were lower than the reference (IIC), and HRs for stages IIIC-IV were higher than the reference, indicating a superior and inferior MSS, respectively. The wide spread of HRs suggests that, once adjusted for patient- and disease-characteristics, the TNM8 staging system appropriately separates prognostically distinct subgroups with respect to MSS. Stage IIIA and IIIB were exceptions. In the case of IIIA, the HR was smaller than the IIC reference group, despite being a higher stage (HR 0.28 [95% Confidence Interval {CI} 0.14–0.56]). In the case of IIIB, the HR was similar in magnitude to the reference group (HR 0.86 [ 95% CI 0.54–1.36]). The HR increased sequentially with each stage from IA-IIC and IIIA-IIID as the MSS declined (Table 3). The introduction of stage IIID led to a reduction in HR for stages IIIA/B/C, with stage IIIC having the largest decrease.

**Table 3:** Adjusted cox regression hazard ratios for melanoma-specific survival of each stage of the 7^th^ and 8^th^ edition TNM melanoma staging system

	TNM7			TNM8		
	Hazard Ratio	95% CI	*P*-value	Hazard Ratio	95% CI	*P*-value
**Stage**						
IA	0.03	(0.02–0.05)	< 0.001	0.04	(0.02–0.06)	< 0.001
IB	0.12	(0.08–0.18)	< 0.001	0.17	(0.11–0.26)	< 0.001
IIA	0.35	(0.24–0.51)	< 0.001	0.33	(0.22–0.48)	< 0.001
IIB	0.66	(0.47–0.93)	0.019	0.66	(0.46–0.93)	0.017
IIC	Reference	-	-	Reference	-	-
IIIA	0.56	(0.35–0.91)	0.020	0.28	(0.14–0.56)	< 0.001
IIIB	0.94	(0.65–1.35)	NS	0.86	(0.54–1.36)	NS
IIIC	1.95	(1.35–2.82)	< 0.001	1.42	(1.02–1.97)	0.040
IIID	-	-	-	4.14	(2.26–7.58)	< 0.001
IV	6.48	(4.04–10.37)	< 0.001	6.57	(4.08–10.57)	< 0.001

Adjusted for report/diagnostic year, age, sex, Elixhauser comorbidity index, neighbourhood income quintiles, place of residence, residential area, histological subtype, location, adjuvant/palliative systemic therapy and adjuvant/palliative radiotherapy

Similarly for OS, there was stepwise separation of prognostically distinct subgroups in most cases (see Additional file 4). Stage IIIA had a lower risk of death than the IIC reference group, similar to MSS findings (HR 0.34 [95% CI 0.20–0.58]). As well, the stage IIIB and IIIC HRs were similar in magnitude to the reference group (HR 0.77 [95% CI 0.52–1.14]; HR 1.19 [95% CI 0.91–1.57]).

### Explanation of variance in survival

For TNM8, 19.7% and 17.2% of the variance for the Cox regression for MSS and OS, respectively, is explained by the staging information. For TNM7, the explained variance remains similar at 18.9% and 16.6% for MSS and OS, respectively.

## Discussion

Our study set out to investigate the performance of the 8^th^ edition UICC/AJCC TNM (Tumour, Nodes, Metastasis) staging system for cutaneous melanoma using a Canadian population-based sample. We believe our study is the largest population-based validation study to date, with 6,414 eligible participants from stage I-IV. Our study used detailed demographic and disease data from the provincial cancer registry and other administrative sources to estimate overall survival, melanoma-specific survival, and cox regression hazard ratios.

The changes made between TNM7 and TNM8 resulted in the reclassification of many patient’s stage subgroups. Stage IIIC was the largest TNM8 stage III subgroup, representing 54.3% of our sample, 48% of the IMDDP, and 41–65% in the literature [6–13]. It is important for providers and patients – in discussion of risks and treatment options – to be aware that stage IIIC is now the most common stage III subgroup and has higher survival than reported for TNM7 stage IIIC. Notably, the newly introduced stage IIID includes no evidence of distant metastasis, yet the prognosis is poor, and survival is overlapping with stage IV metastatic disease. We found that within stage I, there was substantial movement from IB to IA, due to changes in criteria on thickness, ulceration, and mitosis. However, stages I and II had minimal changes in their 2-year and 5-year MSS. This aligns with the literature reporting little or no changes for stages I and II 5-year survival [6–10].

Very few patients shifted between the broad stage groups (I, II, III, IV) with 8 moving from stage II to stage I. This highlights that differences in stage subgroup survival relates overwhelmingly to shifts within a given stage’s subgroups (e.g. shifts between TNM7 IIIB and TNM8 IIIC). Our findings also suggest that historic data staged with TNM7 can be expected to have very similar underlying major disease factors (e.g. thickness, node positivity, presence of distant metastasis) to TNM8 staged patients for broad stage groups. The small improvement in the percent of variance in survival explained by stage is of a similar magnitude to typical observations for other cancer sites. In general, the percent of variance explained is low [21–23].

Survival for melanoma decreases with increasing age, ulceration rate, and male sex. Our median age of 61 (IQR 49–73) aligns with that found in several studies [6, 11, 13]. The sex distribution of our cohort at 51.7% males vs 48.3% females was evenly distributed, and our ulceration rate of 16.84% was lower than several studies (30–50%) [8, 11–13]. However, our study sample is composed of 57.6% TNM8 stage IA; a low ulceration rate is expected and consistent with one other study reporting a 16% ulceration rate and a 60% sample proportion of TNM8 stage IA [7].

While the OCR has an excellent tumour-capture rate, some limitations are present. Ulceration, an important prognostic factor, could not be determined in 6.8% of cases. Moreover, risks of misclassification of cause of death from secondary cancers (not counted in MSS) being related to melanoma metastases, and heterogeneous pathology reporting, all introduce potential sources of error.

We demonstrated lower survival for nearly all stages in comparison to the largely institution-based IMDDP that first established the AJCC8 staging system. Our TNM8 5-year MSS for stage I (98.4%), stage II (82.5%) and stage III (66.4%) was comparable or lower than the 5-year MSS for stage I (98%), stage II (90%), and stage III (77%) reported by *Gershenwald *et al*. *using the IMDDP [4].

Our 5-year MSS estimates were lower than the IMDDP for all stage subgroups except IA, IIIA, and IIID, which contained the IMDDP value in their 95% CI. Our 5-year MSS was lower than the IMDDP for stage IB (95.6% vs 97%), IIA (90.4% vs 94%), IIB (80.8% vs 87%), IIC (69.0% vs 82%), IIIB (74.8% vs 83%), and IIIC (59.0% vs 69%) [4]. Other validation studies (population-based and institution-based) of the TNM8 similarly reported a lower survival than the IMDDP for many substages [6, 8–13].

It is not immediately clear why the IMDDP cohort reported higher survival for most stages. The IMDDP analysis used institutional data of 46,000 patients from ten institutions globally. None of these institutions were Canadian, and 15,746 patients (33.5%) were from three institutions in the U.S. [4], where access to healthcare is influenced by private and public health coverage. As no demographic and disease characteristics were included, it is difficult to ascertain the influence of confounding variables. Disparities in the study population (e.g., young age, unbalanced sex ratio, higher SES) or disease features (e.g., decreased ulceration rate, primary tumour location) may have influenced survival estimates. We note that similar to the IMDDP data, our population-based sample comes predominantly before the adoption of new targeted and immune-based therapies for melanoma. This suggests that differences in use of approved novel targeted therapies and immune-based therapies is less likely to be the primary driver [24]. As such, it is important to recognize that the results from the IMDDP analysis may not be generalizable to Canadian populations, and the institutional dataset may differ from population-based outcomes.

Our findings also emphasize the importance of considering subgroup-specific treatment recommendations, and evaluating novel adjuvant therapies for high-risk stage II patients. We highlight this given that survival for stage III and IV patients is improving with immunotherapies (anti-PD1, anti-CTLA4) and targeted therapies (BRAF inhibitors, MEK inhibitors) [25]. Currently, adjuvant pembrolizumab (anti-PD1), nivolumab (anti-PD1), or combination dabrafenib (BRAF inhibitor) and trametinib (MEK inhibitor) are recommended for resected stage III melanoma (dependant on *BRAF *status) [26, 27]. These same therapies, however, are not recommended for resected stage II melanoma. Notably, stage IIIA has a lower risk of death than stage IIC, and stage IIIB has a similar risk to stage IIC.

Shifts in stage III subgroups are also notable. The MSS for stage IIIC improved between TNM7 and TNM8, and the MSS for stage IIID approximated stage IV metastatic disease. The need for expanded treatment recommendations is clear, and several ongoing phase 3 trials will provide valuable insight (NCT04309409, NCT03553836, NCT04099251). In the future, patients will surely benefit from increased access to innovative therapies and further-refined treatment guidelines.

## Conclusion

We investigated the performance of the 8^th^ edition UICC/AJCC TNM melanoma staging system in the largest population-based sample published to date. We found that the staging system performed well, with good separation of stage subgroups and a small improvement in the percent of variance explained compared to TNM7. We note that our MSS was similar to other validation studies, though inferior to the original institution-based sample that drove the development of the TNM8. This difference is important for clinicians to consider when advising their patients on expected stage-based outcomes.

## Supplementary Information

**Additional file 1: Table S1.** Stage III subgroup reclassification from TNM7 to TNM8. N (row percent).

**Additional file 2: Appendix 1.** Overall survival (OS) for the TNM (A) 7th edition and (B) 8th edition.

**Additional file 3: Table S2.** 2-year and 5-year overall survival and 95% confidence interval (CI) for each stage of the 7th and 8th edition TNM melanoma staging system.

**Additional file 4: Table S3.** Adjusted cox regression hazard ratios for overall survival of each stage of the 7th and 8th edition TNM melanoma staging system.

## Data Availability

The dataset from this study is held securely in coded form at ICES. While legal data sharing agreements between ICES and data providers (e.g., healthcare organizations and government) prohibit ICES from making the dataset publicly available, access may be granted to those who meet pre-specified criteria for confidential access, available at www.ices.on.ca/DAS (email: das@ices.on.ca). The full dataset creation plan and underlying analytic code are available from the authors upon request, understanding that the computer programs may rely upon coding templates or macros that are unique to ICES and are therefore either inaccessible or may require modification.

## Abbreviations

AJCC: American Joint Committee on Cancer
ALR: Activity Level Reporting
CCO: Cancer Care Ontario
CIHI DAD and SDS: Canadian Institute for Health Information Discharge Abstract Database and Same Day Surgery
HR: Hazard ratio
IKN: ICES Key Number
IMDDP: International Melanoma Database and Discovery Platform
IQR: Interquartile range
MSS: Melanoma-specific survival
NDFP: New Drug Funding Program
OCR: Ontario Cancer Registry
ODB: Ontario Drug Benefit
OHIP: Ontario Health Insurance Plan
ORGD: Office of the Registrar General-Deaths
OS: Overall survival
RIO: Rurality Index for Ontario
RPDB: Registered Persons Database
TNM: Tumour, Nodes, Metastasis
UICC: Union for International Cancer Control
95% CI: 95% Confidence interval
