# Supplementary material for: A population-based validation study of the 8th edition UICC/AJCC TNM staging system for cutaneous melanoma
Source: BMC Cancer. 2022 Jul 1;22:720. doi: 10.1186/s12885-022-09781-0 (PMC9248086; doi:10.1186/s12885-022-09781-0)
Supplement: Supplementary file 1 — Additional file 1: Table S1. Stage III subgroup reclassification from TNM7 to TNM8. N (row percent). [file 12885_2022_9781_MOESM1_ESM.pdf]

Supplemental Table 1. Stage III subgroup reclassification from TNM7 to TNM8. N (row percent).

| | | TNM8 | | | | |
|---|---|---|---|---|---|---|
| | | IIIA | IIIB | IIIC | IIID | Total |
| TNM7 | IIIA | 89 (51.7%) | 57 (33.1%) | 26 (15.1%) | 0 | 172 |
| | IIIB | 23 (10.5%) | 54 (24.7%) | 142 (64.8%) | 0 | 219 |
| | IIIC | 0 | 11 (5.3%) | 157 (75.8%) | 39 (18.8%) | 207 |
| | Total | 112 | 122 | 325 | 39 | 598 |
