# Supplementary figures and images for: A population-based validation study of the 8th edition UICC/AJCC TNM staging system for cutaneous melanoma
Source: BMC Cancer. 2022 Jul 1;22:720. doi: 10.1186/s12885-022-09781-0 (PMC9248086; doi:10.1186/s12885-022-09781-0)

(A) Kaplan-Meier Survival Curve

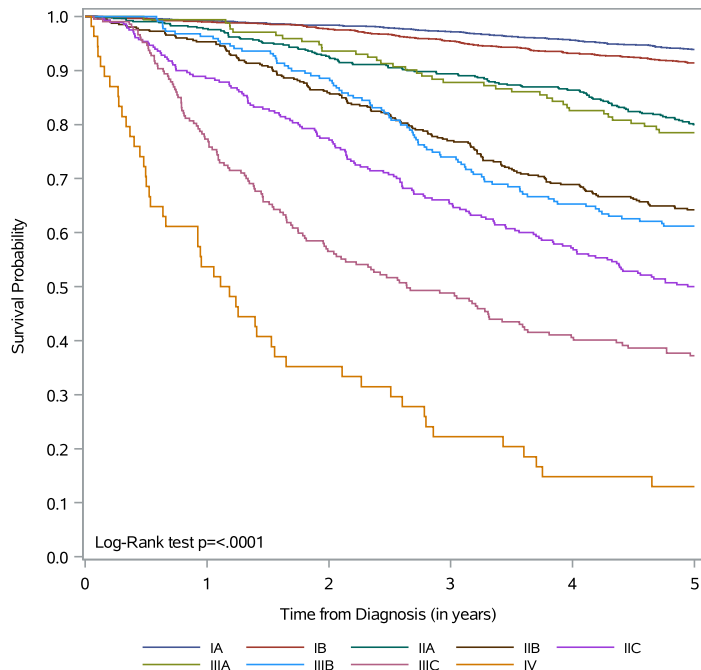

(B) Kaplan-Meier Survival Curve

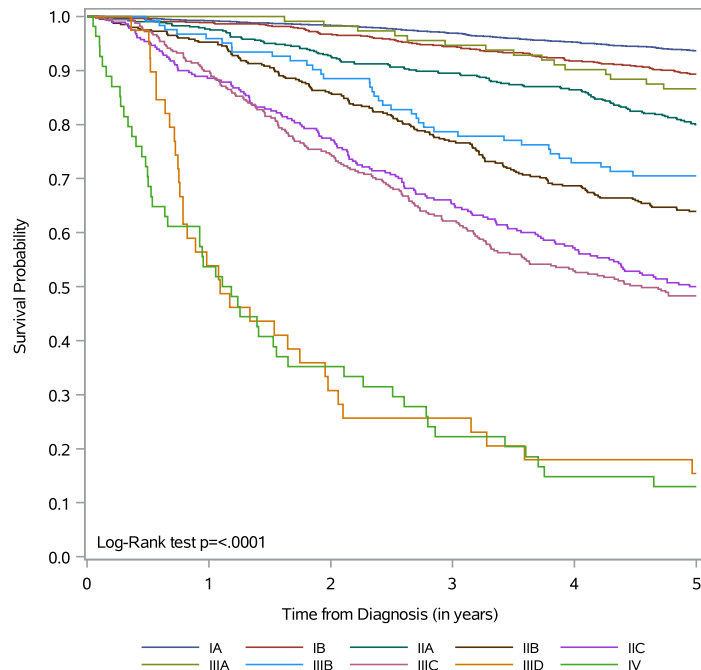

Supplement: Supplementary file 2 — Additional file 2: Appendix 1. Overall survival (OS) for the TNM (A) 7th edition and (B) 8th edition. [file 12885_2022_9781_MOESM2_ESM.pdf]
