# Supplementary material for: A population-based validation study of the 8th edition UICC/AJCC TNM staging system for cutaneous melanoma
Source: BMC Cancer. 2022 Jul 1;22:720. doi: 10.1186/s12885-022-09781-0 (PMC9248086; doi:10.1186/s12885-022-09781-0)
Supplement: Supplementary file 3 — Additional file 3: Table S2. 2-year and 5-year overall survival and 95% confidence interval (CI) for each stage of the 7th and 8th edition TNM melanoma staging system. [file 12885_2022_9781_MOESM3_ESM.pdf]

Supplemental Table 2. 2-year and 5-year overall survival and 95% confidence interval (CI) for each stage of the 7th and 8th edition TNM melanoma staging system.

	Overall Survival							
	TNM7				TNM8			
Stage	2-year	95% CI	5-year	95% CI	2-year	95% CI	5-year	95% CI
IA	98.4%	97.8-98.8%	93.9%	92.9-94.7%	98.4%	97.9-98.7%	93.6%	92.8-94.4%
IB	97.7%	96.9-98.2%	91.4%	90.0-92.6%	96.8%	95.3-97.8%	89.4%	87.1-91.2%
IIA	92.4%	89.8-94.4%	80.0%	76.3-83.1%	92.6%	90.0-94.5%	80.0%	76.3-83.1%
IIB	85.9%	82.1-89.0%	64.2%	59.3-68.7%	85.8%	82.0-88.9%	63.9%	59.0-68.4%
IIC	77.1%	71.8-81.6%	50.0%	44.0-55.7%	77.1%	71.8-81.6%	50.0%	44.0-55.7%
IIIA	93.6%	88.8-96.4%	78.5%	71.6-83.9%	98.2%	93.1-99.6%	86.6%	78.8-91.7%
IIIB	88.6%	83.6-92.1%	61.2%	54.4-67.3%	88.5%	81.4-93.0%	70.5%	61.5-77.7%
IIIC	56.5%	49.5-63.0%	37.2%	30.6-43.7%	74.5%	69.4-78.9%	48.3%	42.8-53.6%
IIID	-	-	-	-	30.8%	17.3-45.4%	15.4%	6.2-28.3%
IV	35.2%	22.8-47.8%	13.0%	5.7-23.3%	35.2%	22.8-47.8%	13.0%	5.7-23.3%
