# Supplementary material for: A population-based validation study of the 8th edition UICC/AJCC TNM staging system for cutaneous melanoma
Source: BMC Cancer. 2022 Jul 1;22:720. doi: 10.1186/s12885-022-09781-0 (PMC9248086; doi:10.1186/s12885-022-09781-0)
Supplement: Supplementary file 4 — Additional file 4: Table S3. Adjusted cox regression hazard ratios for overall survival of each stage of the 7th and 8th edition TNM melanoma staging system. [file 12885_2022_9781_MOESM4_ESM.pdf]

Supplemental Table 3. Adjusted cox regression hazard ratios for overall survival of each stage of the 7th and 8th edition TNM melanoma staging system.

	TNM7			TNM8		
	Hazard Ratio	95% CI	P-value	Hazard Ratio	95% CI	P-value
**Stage**						
IA	0.16	(0.12-0.21)	<0.001	0.16	(0.13-0.21)	<0.001
IB	0.23	(0.18-0.31)	<0.001	0.25	(0.19-0.34)	<0.001
IIA	0.44	(0.34-0.58)	<0.001	0.42	(0.32-0.55)	<0.001
IIB	0.76	(0.59-0.98)	0.032	0.76	(0.59-0.98)	0.033
IIC	Reference	-	-	Reference	-	-
IIIA	0.61	(0.41-0.89)	0.010	0.34	(0.20-0.58)	<0.001
IIIB	0.90	(0.66-1.21)	NS	0.77	(0.52-1.14)	NS
IIIC	1.48	(1.08-2.03)	0.014	1.19	(0.91-1.57)	NS
IIID	-	-	-	4.24	(2.61-6.88)	<0.001
IV	4.20	(2.69-6.57)	<0.001	4.33	(2.74-6.86)	<0.001

Adjusted for report/diagnostic year, age, sex, Elixhauser comorbidity index, neighbourhood income quintiles, place of residence, residential area, histological subtype, location, adjuvant/palliative systemic therapy and adjuvant/palliative radiotherapy.
